# The Principles and Practice of Medicine

**Published:** 1892-05

**Authors:** 


### Book Reviews.

THE PRINCIPLES AND PRACTICE OF MEDICINE.—By William Osler, M. D., Fellow of the Royal College of Physicians, London; Professor of Medicine in the Johns Hopkins University and Physician-in-Chief to the Johns Hopkins Hospital; Formerly Professor of the Institutes of Medicine, McGill University, Montreal, and Professor of Clinical Medicine in the University of Pennsylvania. Published by D. Appleton & Co., New York, 1892.

With the light that has been thrown upon the science of medicine by histological researches and bacteriological investigations, by the additional knowledge upon old remedies and the introduction of newer ones, by the rapid strides that have been made in preventive medicine, and the knowledge gained of climatic influences upon certain diseases, the older textbooks are far in the wake of this advanced period and have somewhat lost their fitness for the student and practitioner of to-day.

This work embraces all the best and most progressive views relative to the practice of medicine, and shows exhaustive research and diligent study by the author, whose wide experience and thorough fitness has placed him in an attitude to give to the profession a work which altogether meets the demand.

To the busy practitioner we commend it as being concise but thorough, to the scientific investigator it will act as a stimulant and a guide to assist in unraveling the tangled meshes and obscure points, and to the student it will serve to steer the untrained mind in the most approved channels.

